# Mushroom Poisoning: A Case Series With a Literature Review of Cases in the Indian Subcontinent

**DOI:** 10.7759/cureus.39550

**Published:** 2023-05-26

**Authors:** Vikas Vaibhav, Raviprakash Meshram, Yashpal S, Nishi Jha, Gitanjali Khorwal

**Affiliations:** 1 Forensic Medicine and Toxicology, All India Institute of Medical Sciences (AIIMS) Rishikesh, Rishikesh, IND; 2 Pathology and Laboratory Medicine, All India Institute of Medical Sciences (AIIMS) Rishikesh, Rishikesh, IND; 3 Anatomy, All India Institute of Medical Sciences (AIIMS) Rishikesh, Rishikesh, IND

**Keywords:** ramaria sanguinea, wild mushroom, mycetism, mushroom poisoning, autopsy

## Abstract

Mushroom is a fungus widely used as an edible in various parts of the world, especially in hilly regions with damp climates. Nevertheless, when picked from the wild to use as a vegetable, it has proved fatal for people ingesting it due to a lack of knowledge for distinguishing between poisonous and non-poisonous mushrooms by the people of the local community. Three cases presented as emergencies from a single household comprising a 13-year-old girl and both her grandparents following the ingestion of mushrooms picked from a nearby forest area. Luckily the parents of the girl were out for work, so they survived and helped identify the mushroom. Most cases are not reported or documented, and data are present mainly in the form of case reports.

## Introduction

A mushroom is a fleshy fruiting body of fungus usually present above ground on soil or its food source [1]. Due to its hallucinogenic properties, it is used as an edible in the form of vegetables, for medicinal purposes, and for intoxication [2]. Nowadays, their increased consumption as a source of food has led to increased incidences of poisoning, which may be possibly hypothesized as difficulty in distinguishing between poisonous and non-poisonous mushrooms by the naked eye, ingestion of nearby toxic species along with edible species, incomplete or improper cooking, and contamination with other microorganisms such as bacteria or viruses [2-6].

## Case presentation

We received three bodies from a family comprising the grandfather (case no 1), grandmother (case no 2), and granddaughter (case no 3) with an alleged history of wild mushroom ingestion picked from the nearby forest in Lambgaon, Tehri-Garhwal region of Uttarakhand. The parents of the girl child were not at home, and hence they did not consume food with mushrooms.

Similarly, the grandmother of the girl was a 61-year-old female with an antecedent history of mushroom ingestion on 12/08/2021 and presented with complaints of frequent episodes of vomiting and loose stools. Given the poor Glasgow Coma Scale (GCS), she was intubated and put on a mechanical ventilator. Investigations showed elevated liver enzymes with coagulopathy with grade 3 encephalopathy (Table 1). Appropriate treatment with antibiotics, anti-edema drugs, inotropes, and anti-epileptic drugs was given. She developed cardiac arrest at 02:20 AM on 21/08/2021, could not be revived, and was declared dead on 21/08/2021 at 02:50 AM (Table 2).

**Table 1:** Clinical and biochemical parameters of case 1. DOA, date of admission; HCV, hepatitis C virus; HIV, human immunodeficiency virus; HBsAg, hepatitis B surface antigen; SarsCoV, severe acute respiratory coronavirus syndrome

Parameters	Case 1	Reference range
Residence	Rural, Garhwal-Himalayan region	
Type of mushroom	Wild	
Symptoms	Vomiting (3-4 episodes), loose stools, epigastric pain, and jaundice	
Day of admission in ICU	3^rd^ DOA	
Day post ingestion	9^th^ day	
GENERAL FEATURES		
Sensorium	Fair, conscious	
Blood pressure	100/68 mmHg	120/80 mmHg
Pulse rate	112/min	60-100/min
Respiratory rate	20/min	12-16/min
Spo2	98%	95%-100%
Temperature	98°F	97°F-99°F
INVESTIGATIONS		
Hemoglobin	15 g/dL	Male: 14-18 g/dL, Female: 12-16 g/dL
Total leucocyte count	14500/µL	4000-10000/µL
Differential leucocyte count	Neutrophil-78%, Lymphocyte-6%, Monocyte-3%, Eosinophil-13%, Basophil-0%	Neutrophil-40-80%, Lymphocyte- 20-40%, Monocyte-2-10%, Eosinophil-1-6%, Basophil- <2%
Platelets	80000/cubic mm	150000/cubic mm- 400000/cubic mm
Prothrombin time/International normalized ratio	>120	Prothrombin time: 11-13.5 /International normalized ratio: 0.8-1.1
Bilirubin (total/direct)	5.36 mg/dL	0.3-1 mg/dL, 0-0.3 mg/dL
Serum glutamic pyruvic transaminase	6315 IU/dL	< 40 IU/dL
Serum glutamic oxaloacetic transaminase	3310 IU/dL	< 40 IU/dL
Alkaline phosphatase	434 IU/dL	Adult: 100-250 IU/dL; Children: 250-770 IU/dL
Urea	32 mg/dL	5-25 mg/dL
Serum creatinine	1.09 mg/dL	0.5-1.3 mg/dL
Na/K (sodium/potassium)	138 mEq/L, 4.6 mEq/L	135-145 mEq/L, 3.8-5.2 mEq/L
Calcium	9.2 mg/dL	9-11 mg/dL
Phosphate	3.1 mg/dL	3.4-4.5 mg/dL
Uric acid	5.5 mg/dL	2.5-6.2 mg/dL
HCV/HIV/HBsAg	Negative	
SARSCoV	Negative	
Urine routine/Microscopic examination	--	

**Table 2:** Clinical and biochemical parameters of case 2. DOA, date of admission; HCV, hepatitis C virus; HIV, human immunodeficiency virus; HBsAg, hepatitis B surface antigen; SarsCoV, severe acute respiratory syndrome coronavirus

Parameters	Case 2	Reference range
Residence	Rural, Garhwal-Himalayan region	
Type of mushroom	Wild	
Symptoms	Vomiting (3-4 episodes), loose stools, epigastric dull aching pain, and jaundice	
Day of admission in ICU	5^th^ DOA	
Day post ingestion	11^th^ day	
GENERAL FEATURES		
Sensorium	Drowsy	
Blood pressure	100/68 mmHg	120/80 mmHg
Pulse rate	70/min	60-100/min
Respiratory rate	20/min	12-16/min
Spo2	98%	95%-100%
Temperature	98°F	97°F-99°F
INVESTIGATIONS		
Hemoglobin	8.13g/dL	Male: 14-18 g/dL, Female: 12-16 g/dL
Total leucocyte count	9100/µL	4000-10000/µL
Differential leucocyte count	82%/6%/3%/12%	Neutrophil 40-80%, Lymphocyte 20%-40%, Monocyte 2%-10%, Eosinophil 1%-6%, Basophil- <2%
Platelets	70000/cubic mm	150000/cubic mm-400000/cubic mm
Prothrombin time/International normalized ratio	--	Prothrombin time: 11-13.5 /International normalized ratio: 0.8-1.1
Bilirubin (Total/Direct)	5.55 mg/dL /2.48 mg/dL	0.3-1 mg/dL, 0-0.3 mg/dL
Serum glutamic pyruvic transaminase	7150 IU/dL	< 40 IU/dL
Serum glutamic oxaloacetic transaminase	4500 IU/dL	< 40 IU/dL
Alkaline phosphatase	560 IU/dL	Adult: 100-250 IU/dL Children: 250-770 IU/dL
Urea	15 mg/dL	5-25 mg/dL
Serum creatinine	2.13 mg/dL	0.5-1.3 mg/dL
Na/K (sodium/potassium)	130 mEq/L, 2 mEq/L	135-145 mEq/L, 3.8-5.2 mEq/L
Calcium	3.8 mg/dL	9-11 mg/dL
Phosphate	--	3.4-4.5 mg/dL
Uric acid	--	2.5-6.2 mg/dL
HCV/HIV/HBsAg	Negative	
SARS CoV	Negative	
Urine Routine/Microscopic Examination	--	

The girl was a 12-year-old with an antecedent history of mushroom ingestion four days back on 12/08/2021 and she presented with complaints of frequent episodes of vomiting (18-20 times per day), loose stools (10-12 episodes) in two days, and altered sensorium for one day.

Given poor GCS, she was intubated and put on a mechanical ventilator. Investigations showed elevated liver enzymes with coagulopathy with grade 3 encephalopathy (Table 1). Appropriate treatment with antibiotics, anti-edema drugs, inotropes, and anti-epileptic drugs was given, but the patient developed cardiac arrest episodes twice, for which she was revived at 12:10 AM. She developed a third episode of cardiac arrest, could not be revived, and was declared dead on 20/08/2021 at 12:30 AM (Table 3).

**Table 3:** Clinical and biochemical parameters of case 3. DOA, date of admission; HCV, hepatitis C virus; HIV, human immunodeficiency virus; HBsAg, hepatitis B surface antigen; SarsCoV, severe acute respiratory syndrome coronavirus

Parameters	Case 3	Reference range
Residence	Rural, Garhwal-Himalayan region	
Type of mushroom	Wild	
Symptoms	Vomiting (18-20/day), loose stools (10-12/day), altered sensorium, and abdominal pain	
Day of admission in ICU	5^th^ DOA	
Day post ingestion	9^th^ day	
GENERAL FEATURES		
Sensorium	Disoriented	
Blood pressure	104/60 mmHg	120/80 mmHg
Pulse rate	126/min	60-100/min
Respiratory rate	26/min	12-16/min
Spo2	98%	95%-100%
Temperature	98°F	97°F-99°F
INVESTIGATIONS		
Hemoglobin	11 g/dL	Male: 14-18 g/dL, Female:12-16 g/dL
Total leucocyte count	11710/µL	4000-10000/µL
Differential leucocyte count	86%/9%/3%/0%/2.2%	Neutrophil 40%-80%, Lymphocyte 20%-40%, Monocyte 2%-10%, Eosinophil 1%-6%, Basophil <2%
Platelets	214000/cubic mm	150000/cubic mm- 400000/cubic mm
Prothrombin time/International normalized ratio	37/3.61	Prothrombin time: 11-13.5 / International normalized ratio: 0.8-1.1
Bilirubin(Total/Direct)	5.36 mg/dL	0.3-1mg/dL, 0-0.3mg/dL
Serum Glutamic Pyruvic Transaminase	6315 IU/dL	< 40 IU/dL
Serum Glutamic Oxaloacetic Transaminase	3310 IU/dL	< 40 IU/dL
Alkaline phosphatase	434 IU/dL	Adult: 100 – 250 IU/dL Children: 250 – 770 IU/dL
Urea	34 mg/dL	5-25 mg/dL
Serum Creatinine	2.09 mg/dL	0.5-1.3 mg/dL
Na/K [Sodium/Potassium]	158 mEq/L, 4 mEq/L	135-145 mEq/L, 3.8-5.2 mEq/L
Calcium	9.1 mg/dL	9-11 mg/dL
Phosphate	10.5 mg/dL	3.4-4.5 mg/dL
Uric acid	--	2.5-6.2 mg/dL
HCV/HIV/HBsAg	Negative	
SARSCoV	Negative	
Urine routine/Microscopic examination	Albumin - 30 mg/dL, RBC - 5 cells/cumm	Albumin - <30 mg/dL, RBC - <3RBC/high power field

**Figure 1:**
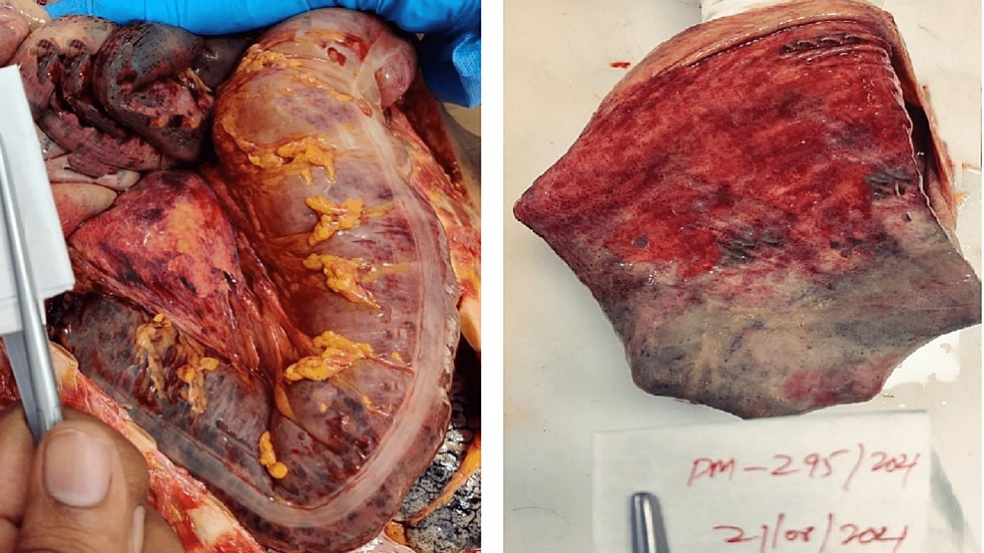
Gross mucosa of the stomach and small intestine showing congestion with blackish discoloration having multiple petechial hemorrhages.

**Figure 2:**
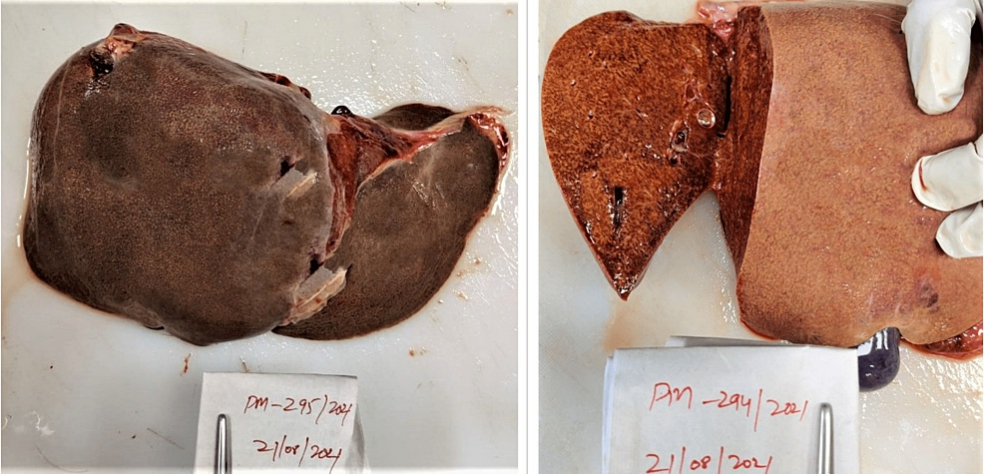
Gross liver with cut section showing nut meg appearance along with yellowish discoloration.

**Figure 3:**
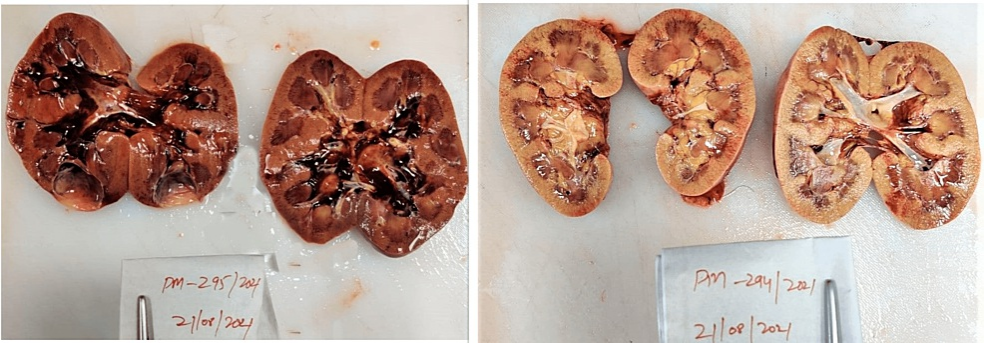
Gross kidneys showing congestion.

Postmortem examination of all the cases showed yellowish discoloration of skin all over the body and sclera. On the reflection scalp, all the cases had yellowish discoloration on the under surface. The brain weighed 1400 g (case 1), 1230 g (case 2), and 1400 g (case 3), respectively; the brain was grossly congested and edematous. On examination of the mucosa of the stomach and small intestine, there was congestion with blackish discoloration having multiple petechial hemorrhages in all three cases (Figure 1). The liver weighed 770 g (case 1), 750 g (case 2), and 770 g (case 3), respectively; the liver was congested (nut meg appearance) in all the cases (Figure 2). The kidneys weighed Left - 170 g, Right - 190 g (case 1), Left - 110 g, Right - 120 g (case 2), Left - 110 g, Right - 80 g (case 3). The kidneys were grossly congested (Figure 3).

**Figure 4:**
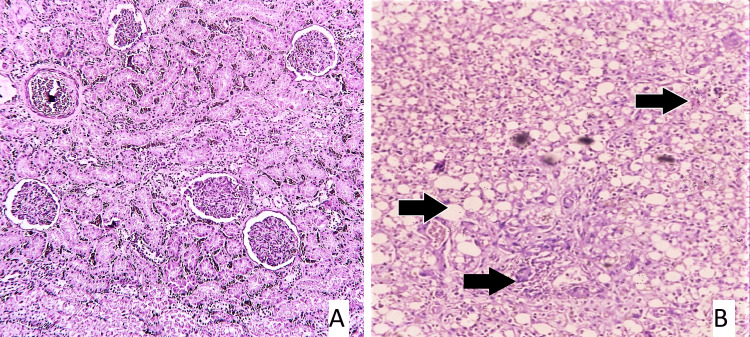
Slide 1 - 20/21 CASE 3 - Slide A: kidney showing normal histology, Slide B: Liver: a - steatosis (arrow), b - ductular proliferation (arrow), c - areas of hemorrhages (arrow) (hematoxylin and eosin stain, magnification - 400x).

**Figure 5:**
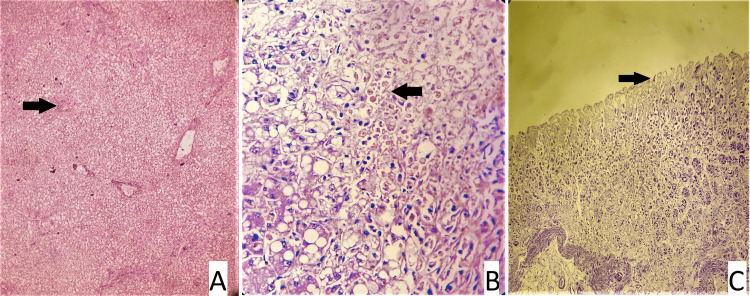
Slide 2: 21/21 CASE 2 - Slide A: liver: a - area of necrosis (arrow), Slide B: kidney: acute tubular necrosis (arrow), Slide C: stomach: mucosal ulceration (arrow) (hematoxylin and eosin stain, magnification - 400x).

**Figure 6:**
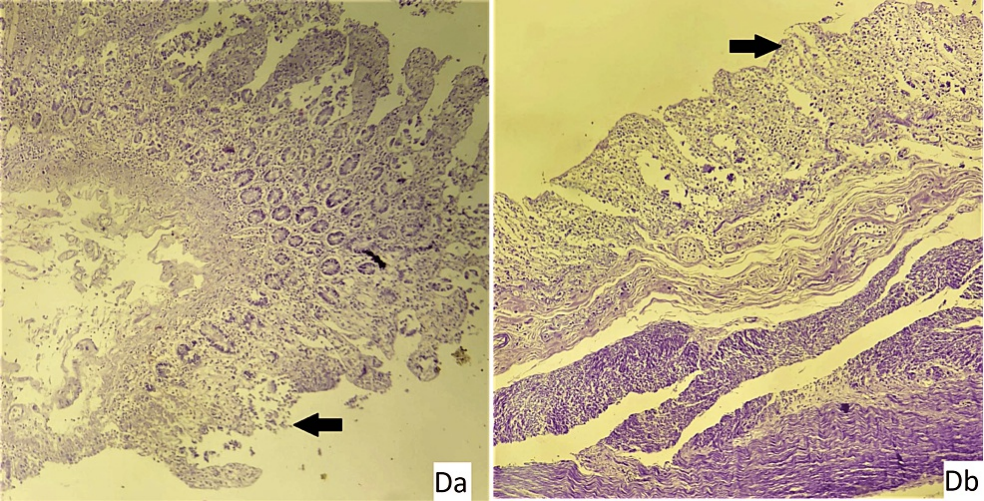
Slide 2: 21/21 CASE 2 - Slide Da: intestine: mucosal ulceration (arrow), Slide Db: intestine: mucosal ulceration (arrow) (hematoxylin and eosin stain, magnification - 400x.

**Figure 7:**
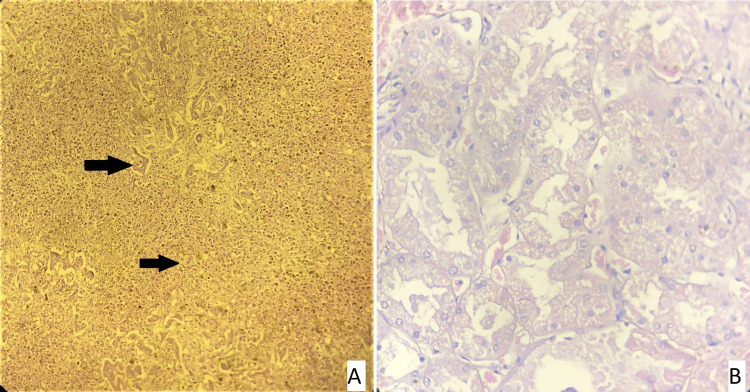
Slide 3: 22/21 CASE - Slide A: liver: a - areas of hemorrhage, b - an area of necrosis, Slide B: kidney showing normal histology, Slide C - stomach mucosa showing autolytic changes, Slide D - intestine showing autolytic changes (hematoxylin and eosin stain, magnification - 400x).

**Figure 8:**
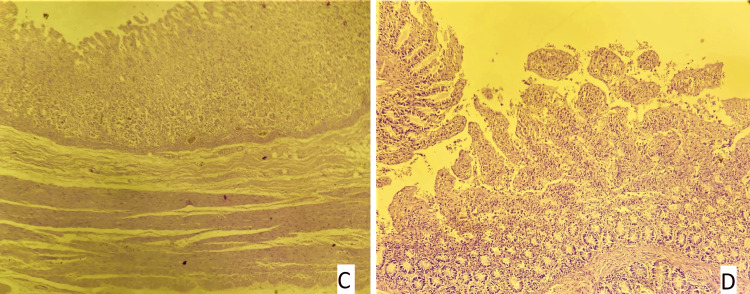
Slide 3: 22/21 CASE - Slide C - stomach mucosa showing autolytic changes (arrow), Slide D - intestine showing autolytic changes (arrow) (hematoxylin and eosin stain, magnification - 400x).

**Figure 9:**
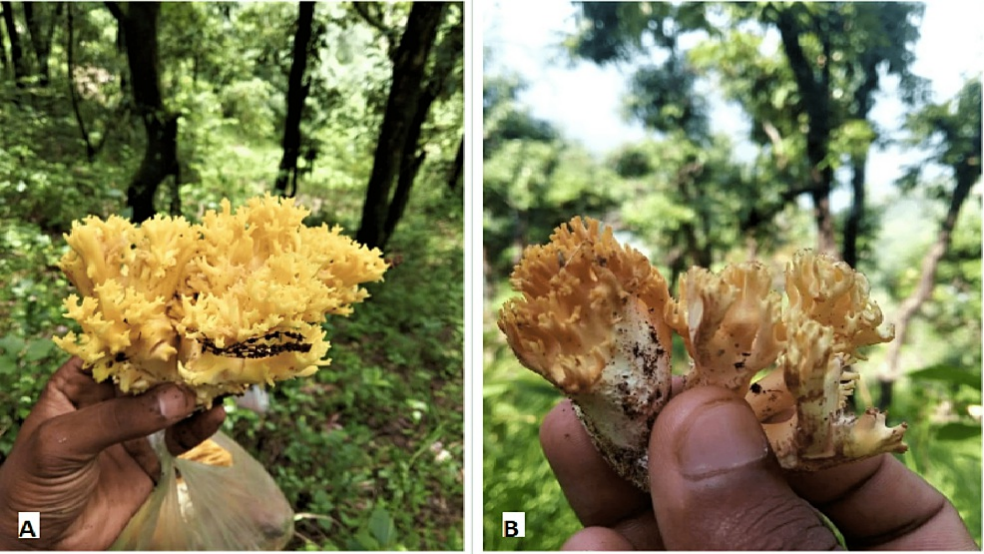
Wild mushrooms that the family consumed.

Following postmortem examination, tissues were retained for histological investigation, and blood and viscera were submitted to the relevant forensic science laboratory (FSL) for chemical analysis. However, the chemical analysis report came negative for toxins. On microscopic examination, liver, kidneys, and intestinal mucosa showed changes suggestive of poisoning, as described in Table 4 and seen in slides 1-3, respectively (Figures 4-9). 

**Table 4:** Histopathological findings in all three cases.

Case 1 (22/21)	Case 2 (21/21)	Case 3 (20/21)
Sections from the liver showed massive necrosis with predominantly zone 3 involvement. In the liver, there were areas of necrosis comprising hemorrhages, hemosiderin-laden macrophages, and apoptotic cells. Kidneys showed normal histology. The mucosa of the stomach, large intestine, and small intestine showed congested capillaries and autolytic changes.	The liver showed zones 2 and 3 necrosis. Kidneys showed features of acute tubular necrosis. The mucosa of the stomach, large intestine, and small intestine showed necrosis and ulceration.	The liver showed intrahepatic cholestasis, macro- and microvesicular steatosis, focal zones 2 and 3 necrosis, and bile ductular proliferation. Areas of old and fresh hemorrhages are identified. Kidneys showed diffuse acute tubular necrosis.

The girl's grandfather was a 63-year-old male who presented with complaints of frequent episodes of vomiting and loose stools. Because of poor GCS, he was intubated and put on a mechanical ventilator. Investigations showed elevated liver enzymes with coagulopathy (Table 1). Appropriate treatment with antibiotics, anti-edema drugs, inotropes, and anti-epileptic drugs was given. He developed cardiac arrest at 03:00 PM on 20/08/2021, could not be revived, and was declared dead on 20/08/2021 at 03:50 PM (Table 1).

## Discussion

Mushroom poisoning is also called 'Cheu-in Uttrakhand.' Ancient works like the "Rigveda" (3500 B.C.) and "Atharvaveda" (1500 B.C.) attest to the fact that mushrooms have been described in humans since the beginning of time [1]. A mother, daughter, and two adult sons were the first people to die from fungus poisoning, and Euripides (456-450 B.C.) remembered the incident with an epigram [1]. Most of the cases in India are from remote villages, especially in Himalayan regions where wild mushrooms are a delicacy. Cases are not reported or if reported, mostly lie in gray literature. There are only case reports published over a period of time [2-6]. There are different outbreaks and epidemio-clinical studies done in different parts of the world [7-9]. People in India are believed to eat roughly 283 types of wild mushrooms within the 2000 species reported worldwide, indicating the vast and profound cultural influence of the majority of Indian ethnic groups [10]. Higher fungi create mushrooms, a fleshy, spore-bearing fruiting body, usually seen above ground on soil or on their food source. About 100 species of mushrooms found in India are poisonous [11]. Amatoxin and Gyromitrin, which are generated by a variety of Amanita species and some members of the Galerina, Lepiota, and Conocybe genera, are the main causes of hepatotoxicity [12-13]. Omphalotus olivascens, Mycena pura, and Chlorophyllum molybdites are frequent poisonous mushroom species found in India, although human poisonings are rare because local ethnic tribes are skilled at distinguishing between toxic and non-poisonous mushrooms [10, 14]. Amanita phalloides poisoning had historically been documented in case series from India, one of which contained 15 cases [14]. Mushroom poisoning can appear clinically as anything from a minor preclinical symptom to a fatal fulminant outcome.

As the toxins in mushrooms do not cause irritation on their own, mushroom poisoning has a lag phase. The absence of any signs or symptoms characterizes the initial phase. It has been observed that the incubation period for amatoxin is comparatively longer than that of other mushroom species. Several species typically start causing gastrointestinal problems much sooner after consumption [15-17].

The second phase also referred to as the gastrointestinal phase, is marked by nausea, vomiting, abdominal cramps, and acute secretory diarrhea. It results in electrolyte imbalances, hypoglycemia, dehydration, and hypotension as well as acid-base problems. At this point, kidney and liver functions are usually normal [15-17].

Then follows the convalescent stage, during which liver failure symptoms may manifest. The effects of toxins continue to harm the liver and kidney, worsening liver and kidney function tests despite improvements in gastrointestinal symptoms [15-17].

Acute liver failure is the final stage, and during this stage, both the liver and the kidneys begin to deteriorate, causing hyperbilirubinemia, coagulopathy, hypoglycemia, acidosis, hepatic encephalopathy, and hepatorenal syndrome. eventually leading to the development of multi-organ failure syndrome, diffuse intravascular coagulation, mesenteric thrombosis, convulsions, and ultimately death [18-19].

All of these patients displayed severe clinical manifestations, including vomiting, diarrhea, jaundice, and hepatic or renal failure within 48 h, or both. Early aspartate aminotransferase (AST)/alanine transaminase (ALT) level increases were strongly correlated with high mortality. The prevalence of mushroom poisoning in children was estimated to be 3.2% of all unintentional poisonings in a prior study by the same organization [20]. The typical clinical manifestation in this case, the autopsy results, and the histological analysis all supported the mushroom poisoning diagnosis. Homicidal poisoning is ruled out by circumstantial evidence.

There is a brief review of cases in the Indian subcontinent due to mushroom poisoning in Table 5.

**Table 5:** Review of literature of cases in the Indian subcontinent due to mushroom poisoning. NA, not applicable; MODS, multiple organ dysfunction syndrome; DIC, disseminated intravascular coagulation; LC-QTOF-MS/MS, liquid chromatography quadrupole time-of-flight mass spectrometry/mass spectrometry; GI, gastrointestinal; AKI, acute kidney injury

Study	No of cases	Relevant findings	Outcome
Barman et al. [20]	3	Renal failure developed in the first case. Accelerated renal failure brought on by Amanita proxima or Amanita smithiana is known to occur and is typically treatable and reversible. The amatoxin-producing mushrooms are likely to be responsible for the last two instances, which showed prominent hepatic involvement and altered liver transaminases, hyperbilirubinemia, and coagulopathy.	One case died of hepatic failure and MODS, and two cases survived.
Greval [21]	12	No work is done to classify mushrooms in India. Most patients present with GI and nervous system symptoms.	Among 12 cases, five survived and seven died.
Garg et al. [22]	3	In the present series the patients were managed with vigorous gastric lavage and activated charcoal, undigested toxins may be removed. The first case died due to hepatotoxicity and two cases survived.	One died due to hepatotoxicity, and two survived.
Verma et al. [23]	4	Early hospitalization, gastric lavage, hydration, penicillin, and silymarin therapy with hepatorenal support are all important components of management because delaying treatment can result in a death rate close to 100%. Cases died due to refractory shock, MODS, sinus bradycardia, ventricular fibrillation, and seizure as terminal events.	One case died on the way, three died in hospital.
Mehta et al. [24]	1	The most prevalent and deadly mushroom toxin that has been identified is amatoxin. Patients who present with acute gastroenteritis during the monsoon season should be evaluated for mushroom poisoning, especially if they are from a region where residents are accustomed to eating wild mushrooms.	The patient passed away as a result, of extensive hepatic dysfunction, which eventually caused organ failure, DIC, and shock.
George and Hegde [4]	4	Four close relatives who accidentally ate deadly mushrooms have developed muscarinic symptoms. They developed toxicity as a result of eating Clitocybe species of mushrooms. Patients who have muscarinic mushroom toxicity present with early-onset symptoms and do well with atropine and supportive therapy for their symptoms.	All cases survived.
Sharma et al. [14]		Fifty-three patients were admitted with mushroom poisoning during the study period of 5 years (2014–2019). The maximum number (16; 30.19%) of the patients belonged to the age group of 11-20 years. A two-and-a-half-month-old baby on breast milk is the youngest patient admitted with mushroom poisoning. Out of 53 patients with mushroom poisoning, 33 (62.26%) were reported in six clusters, and 20 (37.74%) were admitted as individual mushroom poisoning patients. The majority, 40 (75.47%), of the patients survived and got discharged. Nine (16.98%) patients died due to complications of poisoning, and four (7.55%) patients left against medical advice.	Nine died due to complications of poisoning.
Barman et al. [25]	1	Nephrotoxic Amanita ingestion produces gastrointestinal symptoms 20 min-24 h following ingestion, with oliguric renal failure developing within 1-6 days. Mild transaminase elevation is typical. AKI is characterized by interstitial nephritis on renal biopsy and resolves over weeks.	Case survived.
Li et al. [26]	NA	Despite the publication of case studies on edible mushrooms, uncertainty, and ambiguity still exist over whether species are acceptable and safe for consumption. Case reports frequently diverge, and the data proving the claimed qualities of mushrooms may be lacking or unclear.	We investigated 2,786 different types of mushrooms from 99 different nations and 9,783 case reports from more than 1,100 sources. We found 2,189 edible species, of which 2,006 can be eaten without harm, and another 183 species that needed some kind of pretreatment in order to be eaten without harm or that some people may be allergic to. We found 76 unconfirmed species due to unresolved, divergent perspectives on edibility and toxicity, and we identified 471 species with unclear edibility due to missing or insufficient evidence of ingestion.
Kim et al. [27]	1	A 57-year-old male patient was admitted to the emergency room with nausea, vomiting, diarrhea, and abdominal pain. He reported ingesting wild mushrooms with his mother and sister about 2 days ago. His mother and sister were treated with only supportive care, but he was admitted to the intensive care unit and underwent liver transplantation due to acute liver failure. We are reporting a case of fatal Macrolepiota neomastoidea intoxication from wild mushrooms, a rare case of mushroom poisoning.	Survived after liver transplantation.
Yang et al. [28]	1	A 9-year-old boy gradually developed nausea, vomiting, jaundice, and coma within 5 days after ingesting mushrooms. He was treated with conservative care but still deteriorated. On the 7th day after poisoning, he underwent liver transplantation (LT) due to grade IV hepatic encephalopathy. Twenty days later, he recovered and was discharged.	Survived after liver transplantation.
Sun et al. [29]	10	Amanita exitialis is now recognized as an extremely dangerous mushroom. This study reveals that poisoning by A. exitialis is characterized by the long latency before the onset of (6-24 h post-ingestion) GI symptoms and subsequent liver damage characteristic of amatoxin poisoning. A. exitialis is particularly prevalent during the rainy season in subtropical Asia.	Four cases died due to fulminant hepatic failure and six cases survived.
Wang et al. [30]	7	Acute renal failure results from poisoning with Amanita neoovoidea (genus Amanita Pers.). Seven case reports of acute renal failure with acute hepatic failure brought on by ingesting A. neoovoidea are shown here. However, neither phallotoxin nor amatoxin was found in its basidiomata.	All cases survived.
Xu et al. [31]	2	After ingesting wild mushrooms, two individuals showed classic symptoms of muscarinic syndrome. Chills, sweating, salivation, and diarrhea were among the clinical symptoms; the incubation time was roughly two hours. Anti-inflammatory, cleansing, and nutritional assistance were among the restorative treatments. Within 24 h, full recovery started.	All cases survived.
Wang et al. [32]		In China, there have been three cases of severe thrombocytopenia brought on by A. fuliginea poisoning. After ingesting wild A. fuliginea, three patients experienced nausea, vomiting, stomach discomfort, and diarrhea. They all had acute liver damage, coagulopathy, thrombocytopenia (6-41 109/L), and positive fecal occult blood when they first arrived.	Two cases survived, and one died due to fulminant hepatic failure.
Li et al. [33]	2	The scaled white goose cream mushroom that caused poisoning was identified. Liver damage later occurred in two poisoning victims who first experienced gastrointestinal symptoms such as nausea, vomiting, abdominal pain, and diarrhea. One patient was released from the hospital following active rescue and treatment, while the other suffered an acute pulmonary embolism while receiving care. After interventional thrombolysis and further care, he was released.	Both cases survived.
Chu et al. [34]	3	Affected individuals develop a characteristic pattern of whiplike, linear, erythematous wheals within 1-2 days after consumption of raw or cooked shiitake mushrooms. Shiitake dermatitis is self-limited and typically resolves within days to weeks of its appearance.	All cases survived.
Choe et al. [35]	1	In this case, a man drank mushroom liquor with a meal at his home. Seven hours later, he was transported to the emergency room, and 12 h later, he died. This is the first finding of a trichothecene-type mycotoxin in a human biological sample from an expected case of Podostroma cornu-damae intoxication. We demonstrated that LC-QTOF-MS/MS analysis was an effective method for mushroom intoxication cases in the absence of reference materials.	Survived.
Ma et al. [36]	3	Amatoxins are highly toxic and cannot be destroyed by any means of food processing. The liver is the most affected organ, as amatoxins are absorbed preferentially by hepatocytes and go through the enterohepatic circulation. Other organs can also be intoxicated. If the kidneys are involved, acute renal failure secondary to acute tubular necrosis may result.	All three cases survived after liver transplantation.
Sun et al. [37]	1	A patient with Lepiota brunneoincarnata poisoning was admitted to the hospital four days after consuming the mushrooms. Even six days after ingesting L. brunneoincarnata, amatoxins could be found in the bile. After significant hepatotoxicity manifested, the patient recovered with rehydration, endoscopic nasobiliary drainage, and intravenous Legalon SIL infusion.	Survived.
Lin et al. [38]	7	Seven members of one family became ill from Russula subnigricans Hongo species. One person died as a result of their clinical presentations, which ranged from gastrointestinal symptoms to rhabdomyolysis. Early detection and intense supportive care may be crucial to the survival of individuals with rhabdomyolysis induced by R. subnigricans poisoning.	Six cases survived, and one case died due to rhabdomyolysis.
Ahn et al. [39]	2	The fruit body of the unusual fungus P. cornu-damae contains a lethal poison. In this case study, two patients gathered and boiled wild mushrooms in water to consume over the course of a month. Desquamation of the palms and soles, pancytopenia, acute sepsis, and multiple organ failure were seen in the patient who died. After receiving conservative care for a month following admission, the other patient recovered.	One case survived, and one case died due to sepsis and MODS.
Sun et al. [40]	1	R. japonica is a non-lethal gastroenteritis-type poisonous mushroom with mild toxicity and a good prognosis. This article retrospectively analyzed the clinical data of a patient with gastrointestinal bleeding caused by Japanese red mushroom poisoning admitted to the First Affiliated Hospital of Wenzhou Medical University in July 2019. After active hemostasis, stomach protection, fluid replacement, and anti-inflammatory treatment, he got better and was discharged.	Survived.
Bu et al. [41]	1	A 67-year-old female patient with liver failure caused by fatal amanita poisoning due to abdominal pain, vomiting, and diarrhea after eating 350-400 g of Amanita mushroom for 2 days, accompanied by fatigue for 1 day. According to the mechanism of amanita toxin poisoning as enterohepatic circulation, endoscopic retrograde cholangiopancreatography and ultrasound-guided gallbladder puncture and drainage for drainage of bile to discharge toxins were performed to interrupt the enterohepatic circulation of toxins. However, both methods failed, so an open cholecystostomy was performed.	Survived.
Gonmori and Yokoyama [42]	1	Nine prefectures in Japan reported 59 instances of acute encephalopathy in the autumn of 2004 (24 from Akita Prefecture, with eight deaths; age range, 48-93; average age, 70; females, 14, males, 10). Twenty of the 24 cases (20) had kidney disease. Four poisoned participants displayed no kidney issues. Of the 24 cases of poisoning, Pleurocybella porrigens was consumed by 23 victims and Grifola frondosa by one female, in her late 40s who had been on hemodialysis for almost 35 years. She experienced tinnitus, a headache, and vertigo in August. She requested a stay at the hospital after visiting. She consumed 5 g of stewed G. frondosa and 10 g of the same mushroom cooked with chicken and taro on various days while in the hospital. After eating, she experienced pains, lost consciousness, and fell 14-18 days later. Her coma and cramps persisted for almost 10 days, almost till her demise. We came to the conclusion that cyanogenic fungi such as P. porrigens, G. frondosa, Pleurotus eringii, etc. were the source of the encephalopathy that was observed in Akita Prefecture.	Case died.
Musha et al. [43]	5	There have been five incidences of poisoning caused by the local mushroom Hikageshibiretake (Psilocybe argentipes). Since this mushroom contains psilocybin, its clinical effects were generally comparable to those of pure psilocybin. One instance was an acute toxic stupor that culminated in total amnesia, one case involved a psychedelic experience and dreamy consciousness, whereas the other three involved psychotic adverse responses and strong visual hallucinations while conscious. Subjective experiences were accompanied by feelings of worry and terror.	All cases survived.
Lee et al. [44]	4	There were very few cases of mushroom poisoning found every year in Taiwan yet no fatal incidents have been reported. A species named Amanita phalloides had a high lethality rate (22%-33%). We present four children with gastrointestinal syndrome after ingesting wild mushrooms.	All cases survived.
Chaiear et al. [45]	5	The authors describe here five deadly poisoning incidents caused by Amanita virosa that happened to a family that lived in the northeast of Thailand and who, like countless others, had a passion for mushroom hunting. All had passed away from acute liver failure with subsequent hepatoencephalopathy within 4-6 days of consuming the mushrooms.	All cases died of hepatic failure.
Iwafuchi et al. [46]	1	A 66-year-old man with diabetes developed acute renal failure after ingestion of Amanita pseudoporphyria Hongo. Laboratory data showed acute nonoliguric renal failure. A renal biopsy showed acute tubular necrosis with glomerular minor abnormalities. He received hemodialysis treatment for 3 weeks and his renal function normalized 2 months after admission.	Case survived.
Zhang and Huang [47]	19	We report on 19 autopsy cases in China in which the cause of death was poisoning by toxic plants. The emphasis is on analyses of the target organs or tissues affected by these plants. The mechanism of poisoning and cause of death are approached on the basis of the pathologic changes, and associated problems.	All cases died.
Yoon et al. [48]	2	In northeast Asia, the Ganoderma species of mushrooms are consumed as herbal medicine. Following the consumption of the decoction of Ganoderma neojaponicum Imazeki, two cases of concurrent reversible pancytopenia are presented. After receiving conservative care, the patients entirely recovered. Many times, users of herbal medications are unaware of their unwanted effects. Before consuming Ganoderma, patients should be informed of any potential negative effects.	All cases survived.
Yokoyama and Gonmori [49]	NA	Chlorophyllum molybdites, a toxic tropical mushroom, recently invaded and spread throughout central and southern Japan.	NA.

## Conclusions

The most prevalent and poisonous fungi are Amatoxin. Patients who present with acute gastroenteritis during the monsoon season should be evaluated for mushroom poisoning, especially if they are from a region where residents are known to consume wild mushrooms -- all three cases from the family presented with GI symptoms predominantly. Features of acute liver failure along with diffuse acute tubular necrosis, and hemorrhages in the mucosa of the stomach and small intestine having necrosis and ulceration at places were observed histopathologically. Since there is no specific antidote for such poisoning, stopping this recurring issue is essential. People should be aware of such dangerous wild mushrooms and make it a point not to consume them when their source and suitability for consumption are unknown. Early diagnosis and prompt management can save lives.
